# Impact of the COVID-19 Pandemic on Emergency Department Transfers to a Higher Level of Care

**DOI:** 10.5811/westjem.2021.3.50907

**Published:** 2021-05-17

**Authors:** Stephen Lee, Anthony Santarelli, Heesun Choi, John Ashurst

**Affiliations:** * Rocky Vista University College of Osteopathic Medicine, Parker, Colorado; † Kingman Regional Medical Center, Departments of Graduate Medical Education, Kingman, Arizona; ‡ Kingman Regional Medical Center Emergency Medicine, Kingman, Arizona

## Abstract

**Introduction:**

During the coronavirus disease 2019 (COVID-19) pandemic, a reduction in emergency department (ED) visits was seen nationally according to the US Centers for Disease Control and Prevention. However, no data currently exists for the impact of ED transfers to a higher level of care during this same time period. The primary objective of the study was to determine whether the COVID-19 pandemic affected the rate of non-COVID-19 transfers from a rural community ED.

**Methods:**

We completed a retrospective chart review of all ED patients who presented to Kingman Regional Medical Center in Kingman, Arizona, from March 1–June 31, 2019 and March 1–June 31, 2020. To ensure changes were not due to seasonal trends, we examined transfer rates from the same four-month period in 2019 and 2020. Patients were included in the study if they were transferred to an outside facility for a higher level of care not related to COVID-19.

**Results:**

Between the time periods studied there was a 25.33% (P = 0.001) reduction in total ED volume and a 21.44% (P = 0.009) reduction in ED transfers to a higher level of care. No statistical difference was noted in ED transfer volume following adjustment for decreased ED volumes. Transfers for gastroenterology (45%; P = 0.021), neurosurgery (29.2%; P = 0.029), neurology (76.3%; P < 0.001), trauma (37.5%; P = 0.039), urology (41.8%; P = 0.012), and surgery (56.3%; P = 0.028) all experienced a decrease in transfer rates during the time period studied. When gender was considered, males exhibited an increased rate of transfers to psychiatric facilities (P = 0.018).

**Conclusion:**

Significant reductions in both ED volume and transfers have coincided with the emergence of the COVID-19 pandemic. Further research is needed to determine how the current pandemic has affected patient care.

## INTRODUCTION

Emergency physicians determine a patient’s disposition following an initial workup that may include laboratory tests, imaging, and consultation. Once a patient’s disposition has been decided it is crucial to determine whether the patient can be cared for at the current facility or requires transfer to a higher level of care. The need to transfer a patient to a higher level of care is dependent upon numerous factors including availability of specialists, hospital policies, and specialized treatment algorithms.

As cases of the novel coronavirus disease 2019 (COVID-19) increased across the globe, a sharp decrease in emergency department (ED) volumes was noted for various chief complaints while an increase was seen in out-of-hospital cardiac arrests.^1^–^5^ As cases of COVID-19 began to climb in the United States, a 42% reduction in ED volume was reported by the National Syndromic Surveillance Program between March 29–April 25, 2020.^6^ The steepest decrease was noted in females, pediatric patients, and those who lived in the Northeast.^6^ However, data is lacking for those patients who were transferred to a higher level of care after ED evaluation during the current pandemic. In this study we sought to determine whether the rates of non-COVID-19 transfers from a rural ED varied alongside the 2020 COVID-19 pandemic.

## METHODS

### Setting

Kingman Regional Medical Center is located in Mohave County in northern Arizona. Average ED volume ranges between 45,000–60,000 patients annually. The ED houses an Accreditation Council for Graduate Medical Education-accredited emergency medicine residency program that has 18 total trainees.

### Study Design

Following institutional review board approval, we completed a retrospective chart review of all ED patients who were transferred to a higher level of care from March 1–June 31, 2019, and March 1–June 31, 2020. To ensure changes were not due to seasonal trends, we examined transfer rates from the same four-month period in 2019 and 2020. Patients were included in the study if they were transferred to an outside facility for a higher level of care not related to COVID-19. Data were manually abstracted from electronic health records with the use of a quality-controlled protocol and structured abstraction tool that relied on a priori variable selection, systematic abstractor monitoring, and independent verification.^7^

### Statistical Analysis

We conducted all analyses with SPSS Statistics 27 software (IBM Corporation, Armonk, NY). We compared ED and transfer volumes from March–June 2019 with ED and transfer volumes from March–June 2020 with a series of two (year) by two (gender) univariate analysis of variance. Significant interactions and main effects were followed by independent-samples t-tests.

## RESULTS

From March–June 2019 a total of 16,735 patients presented to the ED and there were 691 transfers to a higher level of care. From March–June 2020 there were 13,147 ED patients and 516 transfers. There was an overall 25.33% reduction in average monthly ED patients (*P* = 0.001) and a 21.44% reduction in average monthly ED transfers in 2020 compared to 2019 (*P* = 0.009). However, the average number of patients transferred each month adjusted for ED volume did not vary from 2019 to 2020 (*P* = 0.595).

**Population Health Research Capsule**

What do we already know about this issue?

*During the COVID-19 pandemic, emergency departments (ED) throughout the US saw a sharp reduction in patient volumes; however, the impact on ED transfer rates is unknown.*

What was the research question?

*What affect did the COVID-19 pandemic have on the rate of non-COVID-19 transfers from a rural community ED?*

What was the major finding of the study?

*Significant reductions in both ED volume and transfers have coincided with the emergence of the COVID-19 pandemic.*

How does this improve population health?

*Evaluating the impact of a pandemic on transfer volumes will aid emergency physicians to determine best practices for patient care following initial stabilization.*

When assessed by specialty, there was a reduction in transfers to gastroenterology (45%; *P* = 0.021), neurosurgery (29.2%; *P* = 0.029), neurology (76.3%; *P*<0.001), trauma (37.5%; *P* = 0.039), urology (41.8%; *P* = 0.012), and surgery (56.3%; *P* = 0.028) during the time studied (Figure). A significant interaction between year and gender for the average number of monthly transfers to psychiatry (*P* = 0.035) and surgery (*P* = 0.028) was also detected (Table). Males exhibited an increased rate of transfers to psychiatric facilities from 2019 to 2020 (*P* = 0.018) and females did not (*P* = 0.730). Males also exhibited a decreased rate of transfers for specialized surgical care (*P* = 0.017) and females did not (*P* = 1.00) during the time period studied.

## DISCUSSION

Compared to the same four-month period in 2019, we observed significantly fewer patient visits to a community ED during the peak of the COVID-19 pandemic in Mohave County. This finding aligns with the reduction observed across the US and reported on previously.^6^,^8^–^9^ Additionally, a proportional reduction in specialty-wide transfers to a higher level facility was also seen, indicating that a larger than usual number of individuals in the community setting chose to forgo emergency medical care and definitive specialist treatment. This has been reported in the previous literature where patients with acute cerebral vascular accidents and acute coronary syndrome experienced a decrease in hospital admissions or delayed presentation due to fear of contracting COVID-19.^10^

Although the observed reduction specialty-wide aligned with ED volume, transfers for individual specialty varied considerably in frequency from the 2019 comparison. Transfers to gastroenterology, neurosurgery, neurology, trauma, and urology, matched in volume to the total number of ED patients. Alongside this, transfer rates for vascular, ear nose and throat/oral and maxillofacial surgery, pediatrics, burn, and orthopedics remained low, indicating that irrespective of the emergent disease process the risk of contracting COVID-19 was deemed too high to seek medical care by the patient.

No yearly change in the number of psychiatric transfers was seen, but an increase in the number of men transitioned to a higher level of psychiatric care was noted. Alongside the emergence of the COVID-19 pandemic, an increased prevalence of anxiety, depression, and stress in the US has been reported.^11^,^12^ Although this increase worldwide has been reported to be greater among women, men frequently have more robust resilience mechanisms to resist abrupt changes in mental health.^10^–^14^ As we saw an increase in men requiring transfers due to psychiatric concern, it is postulated that the social climate around the COVID-19 pandemic may uniquely act to diminish resiliency mechanisms in men due to increased stressors and social isolation.

## LIMITATIONS

Retrospective data was collected from a single health network’s ED in northern Arizona and may not be generalizable to all community hospitals across the nation. Although attempts were made to minimize confounding variables by examining a similar time period from the previous year, the rate of ED transfers could have been impacted by specialist coverage and any changes in institutional policies during the time studied. Transfer rates may have also been affected by receiving-facility protocols during the early stages of the pandemic and patient willingness to be transferred to an outside facility.

## CONCLUSION

Significant reductions in both ED visits and transfers have coincided with the emergence of the COVID-19 pandemic in a rural setting. Unlike the rest of ED transfers, males requiring psychiatric care appeared uniquely affected. Further research into how the pandemic affected this patient population is needed.

## Figures and Tables

**Figure:**
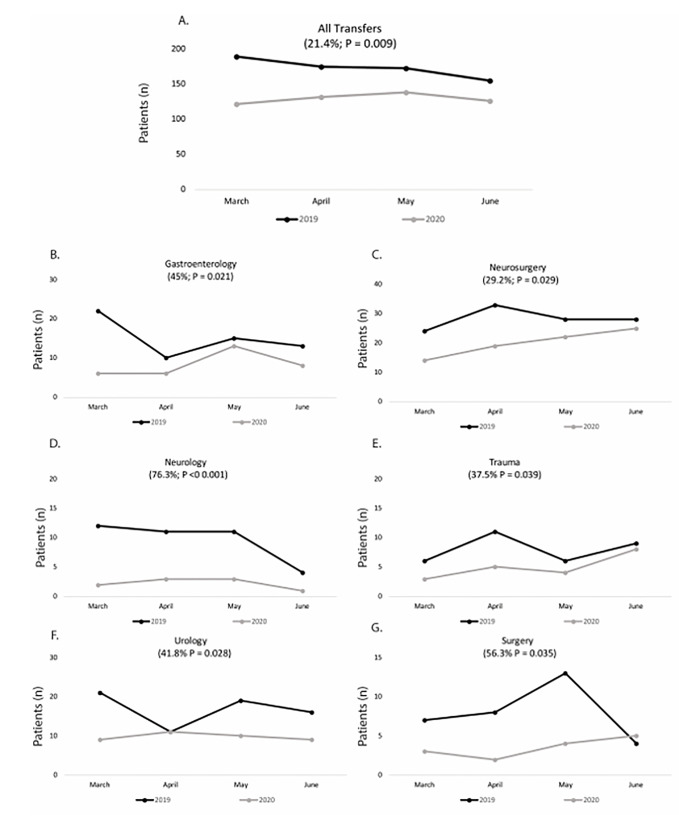
Total monthly transfers to a higher level of care (A). Transfers to individual departments where 2019 and 2020 differ in volume are represented in B–G.

**Table:** Average monthly transfers before and during the COVID-19 pandemic. Data shows the average monthly transfer counts + one standard deviation.

Transfers (Monthly Average)†‡	2019 (03/01–06/31)		2020 (03/01–06/31)	
	
	Females transfered 2019 84.8 + 6.9	Males transfered 2019 88.3 + 10.1	Females transfered 2020 58.5 + 4.5	Males transfered 2020 70.8 + 2.8
Burn	0.8 + 1.0	1.3 + 1.3	1.0 + 1.2	1.5 + 1.0
GI†	8.8 + 4.1	6.3 + 1.3	3.8 + 2.1	4.5 + 1.7
Psychiatry*	23.5 + 2.6	21.8 + 3.1	22.8 + 3.2	27.5 + 1.7
Neurosurgery†	13.5 + 5.3	14.8 + 1.7	8.3 + 2.9	11.8 + 2.2
Neurology†	4.8 + 1.9	4.8 + 2.1	1.0 + 1.2	1.3 + 0.5
Ophthalmology	1.5 + 0.6	1.5 + 1.3	1.3 + 1.3	0.3 + 0.5
Orthopedics	1.0 + 1.4	1.8 + 1.0	1.0 + 1.4	1.0 + 0.8
Trauma†	3.5 + 1.7	4.5 + 1.7	1.8 + 1.0	2.8 + 1.5
Urology†	8.8 + 3.1	8.0 + 2.8	4.8 + 1.5	5.0 + 1.6
Vascular	4.3 + 1.7	5.3 + 1.3	2.5 + 1.7	3.3 + 2.2
ENT/OMFS	3.0 + 1.2	2.0 + 0.8	1.3 + 0.5	2.3 + 2.5
Surgery (non-trauma)*†	3.0 + 2.2	5.0 + 2.7	3.0 + 0.8	0.5 + 0.6
Pediatrics	4.0 + 2.8	6.8 + 2.2	2.5 + 1.9	5.3 + 1.0
Other	4.5 + 1.3	4.8 + 2.4	3.8 + 3.1	4.0 + 2.3
Total Transfers, both genders combined	692		517	

† indicates a significant (P<0.05) main effect of year.

‡ indicates a significant (P < 0.05) main effect of gender.

* indicates a significant (P < 0.05) interaction between year and gender.

*GI*, gastroenterology; *ENT*, ear, nose and throat; *OMFS*, oral and maxillofacial surgery.
